# Mixed Tumours of the Salivary Glands

**Published:** 1904-04-02

**Authors:** 


### MIXED TUMOURS OF THE SALIVARY GLANDS.

The greater number of tumours arising in connection with the salivary glands are of a complex structure, containing as they do a variety of tissues, *e.g.* cartilage, myxomatous tissue, and fat. Besides these definitely mesoblastic structures they contain cells which may resemble epithelial, endothelial, or connective-tissue cells, and accordingly the tumours have been regarded as of epithelial, endothelial, or sarcomatous nature. Hence it will be perceived that there is a wide divergence of opinion among pathologists with regard to the true nature of these peculiar new growths. Dr. F. C. Wood[1] has made a careful study of the clinical histories of a large number of cases of tumour formation in connection with the salivary glands, together with details of the microscopic appearances observed in the tumours after their removal. Of the total number (59), 54 he states may be considered as undoubted mixed tumours of the endothelial type. Only four of these were from the lip, four from the pharynx or palate, two from the neck, and one from the cheek. The remainder were from the parotid or submaxillary glands.

### General Morphology.

The mixed tumours of the salivary glands are found, as a rule, to be encapsulated lobular growths, with harder and softer areas, the denser portions being due, as a rule, to the presence of cartilage or firm connective tissue. They can be divided, roughly, into three groups, each having to a certain extent, characteristic morphology and definite clinical course:—(1) Very fibrous tumours with but little cellular structure, and with but little mucous degeneration and no cartilage; (2) Very hard tumours containing large masses of cartilage and but little connective tissue or cellular parenchyma; (3) Soft, very cellular growths with transparent trabeculæ of mucous tissue surrounding areas, which are opaque and yellow, which on microscopical examination will be found to be dense cellular areas, the colour being occasionally, though not always, due to fatty degeneration or necrosis of the cells. The first and second forms are likely to be benign in their clinical course, while the third form is apt to recur locally or to run an exceedingly malignant course. A few only of the tumours examined belong to the first class, about one-fourth fall into the second group, while the majority belong to the third. It is important to remember, however, that a great amount of variation is present in different parts of the same tumour, and while one portion may be cellular another may be composed largely of cartilage.

### Time of Occurrence.

The development of these tumours may take place before birth, in which case they are often associated with other congenital defects of the facial region. As a rule, the actual development becomes noticeable during early adult life. The average duration of time between the first discovery of the tumour and operation was eight years and nine months in Dr. Wood's cases. In one the tumour had been present for 53 years when it became malignant.

### Situation of Mixed Salivary Tumours.

The growths are seen in the pharynx attached to the bony structures of the hard palate, often indenting the substance of the bone, but not involving the bone substance, from which they are separated by their own fibrous capsule and the periosteum. They also occur on the inner surface of the cheek, usually near the opening of the parotid duct, and in the substance of the lip, most often near the middle line. They are also found in the cervical region in the upper triangle of the neck, in front of the sterno-mastoid muscle. The most frequent situation is near or in the substance of the parotid and submaxillary glands. In the case of large growths the remains of the glandular tissue are stretched out over the tissue of the tumour and undergo pressure atrophy, but are separated from the tumour substance by a capsule. In the pharynx, lips, and cheeks the tumours usually lie just below the surface of the mucous membrane; but the parotid, submaxillary, and cervical new growths are subjacent to the deep fascia. Unless they are large, there is usually considerable mobility. Fixation, if not merely due to the size, is a sign of malignancy. The skin is freely movable over the benign growths.

### Clinical Course.

Out of 37 cases in which the after history is recorded, 20, or 55 per cent., were permanently cured by operation; 17, or 45 per cent., recurred locally. In four of these cases fatal recurrence occurred, and in one other there was a secondary growth too extensive for removal. In the remaining 12 there were local recurrences which were satisfactorily dealt with by further operation. Malignancy can be estimated to a certain extent by the rapidity of growth of the tumours and their physical characters, the softer ones being more likely to be malignant; but it not unfrequently happens that a growth which has existed for a long time without showing any signs of malignancy suddenly takes on a rapid growth, infiltrates surrounding tissues, and forms metastases, though it is very seldom that these latter are found in the lymphatic glands.

Dr. Wood concludes his paper with a summary and critical review of the various theories which have been held by pathologists to account for these growths. He himself inclines to the view that they arise in connection with developmental tissue inclusions derived from the branchial arches, and points out in support of this view that where complicated processes of development occur, there especially are complex tumours found, *e.g.* in kidney, ovary, testicle, and the site of the post-anal gut.

